# Lived experience of persons with multiple sclerosis: A qualitative interview study

**DOI:** 10.1002/brb3.3104

**Published:** 2023-05-29

**Authors:** Sofia Persson, Ann‐Christine Andersson, Boel Andersson Gäre, Bertil Lindenfalk, Jonas Lind

**Affiliations:** ^1^ Jönköping Academy for Improvement of Health and Welfare, School of Health and Welfare Jönköping University Jönköping Sweden; ^2^ Futurum Academy for Health and Care Jönköping Sweden; ^3^ Department of Care Science Malmö University Malmö Sweden; ^4^ Section of Neurology, Department of Internal Medicine County Hospital Ryhov Jönköping Sweden; ^5^ Division of Neurobiology, Department of Biomedical and Clinical Sciences Linköping University Sweden

**Keywords:** coproduction, multiple sclerosis care, patient experience, quality improvement

## Abstract

**Introduction:**

Multiple sclerosis (MS) is a chronic autoimmune disease with a substantial impact on quality of life and functional capability. The prognosis of MS has changed over time due to the development of increasingly effective therapies. As the knowledge and perceptions of persons living with chronic conditions increasingly have been acknowledged, it has become important to understand lived experiences with a focus on everyday events and experiences as a way of knowing and interpreting the world. Exploring context‐specific lived experiences as a source of knowledge about the disease and care may contribute to more precision in designing care services. The aim of this study was to explore the lived experience of persons living with MS in a Swedish context.

**Materials and methods:**

A qualitative interview study was conducted with both purposeful and random sampling strategies, resulting in 10 interviews. Data were analyzed using inductive thematic content analysis.

**Results:**

The analysis generated 4 overarching themes with 12 subthemes, the 4 themes were: perspectives on life and health, influence on everyday life, relations with healthcare, and shared healthcare processes. The themes are concerned with the patients’ own perspectives and context as well as medical and healthcare‐related perspectives. Patterns of shared experiences were found, for example, in the diagnosis confirmation, future perspectives, and planning and coordination. More diverse experiences appeared concerning relations with others, one's individual requirements, symptoms and consequences, and knowledge building.

**Conclusion:**

The findings suggest a need for a more diverse and coproduced development of healthcare services to meet diverse needs in the population with greater acknowledgement of the person's lived experience, including consideration of the complexity of the disease, personal integrity, and different ways of knowing. Findings from this study will be further explored together with other quantitative and qualitative data.

## Graphical abstract

This study provides information on patterns of lived experiences in a population with multiple sclerosis in a Swedish context.The findings emphasize the importance of both standardized and individualized healthcare and underline the value of coproduction in the development of healthcare services.

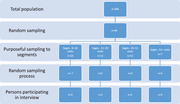

## 1 INTRODUCTION

Multiple sclerosis (MS) is a chronic autoimmune disease affecting 2−400 persons per 100,000 in different populations (Walton et al., 2020). MS prevalence in Sweden is 189/100,000 which is among the highest nationwide prevalence estimates in the world (Ahlgren et al., 2011). The disease targets the central nervous system, leading to loss of neurological functions (Browne et al., 2014; Nelson et al., 2019; Thompson et al., 2018). MS has a substantial impact on the person's quality of life and functional capability (Chruzander et al., 2015; Isaksson et al., 2005), which can diminish income‐earning ability and impose a financial burden on patients and their families (Owens, 2016). MS treatments have changed rapidly through the development of increasingly effective therapies, which have improved prospects for lives free from disability (Tanasescu et al., 2014). Over the past 25 years, life expectancy for people with MS has increased (Kingwell et al., 2012; Lunde et al., 2017; Marrie, 2019). Chronic conditions, such as MS, differ from acute or temporary conditions in relation to the patient's knowledge over time, underlining the importance of patients and health professionals sharing complementary knowledge (Holman & Lorig, 2004). This acknowledgement of the patient's role and knowledge has highlighted the importance of the patient's lived experience (Gaille, 2019; Goulden & Faber, 2020; Irvine, 2016; Mcintosh & Wright, 2019). Lived experience has been described as the choices made and the knowledge gained by a given person and acknowledges aspects of a person's life and identity with a focus on everyday events and experiences as a way of knowing and interpreting the world (Given, 2008). The person's lived experience is also a source of knowledge about the disease and about the medical care received, which in turn may contribute to tailoring medical care (Given, 2008), to assessing the quality of care (Gaille, 2019), and to working on quality improvement of healthcare services (Lachman et al., 2021). In this study, lived experience refers to a person's view of their own health and everyday life, including interactions with healthcare services. Lived experience requires an understanding of specific medical contexts and different economic, social, institutional, and cultural contexts that can illuminate different aspects of a person's life (Gaille, 2019). Previous studies on experiences of persons living with MS in different contexts and healthcare settings identified the burden of illness and the progressive variation in care needs as two important factors that require care institutions to adapt care and support services over time (Borreani et al., 2014; Galushko et al., 2014; Hunter et al., 2021; Methley et al., 2017). To contribute to more precision in care services, there is a need for more individual and person‐centered approaches (Borreani et al., 2014; Galushko et al., 2014; Morley et al., 2013). Coproduction between patients and professionals in quality improvement of healthcare services is suggested to be an opportunity (Lachman et al., 2021). In the medical context of MS—with rapid medical development (Tanasescu et al., 2014), considerable differences in the utilization of total care and cost (Lind et al., 2022), and the increased awareness of the patient's role and knowledge in chronic illness (Gaille, 2019; Goulden & Faber, 2020; Irvine, 2016; Mcintosh & Wright, 2019)—studies of lived experiences in different contexts and populations over time are important. This study takes place in a Swedish Region with a tradition of quality improvement (Bodenheimer et al., 2007; Gäre & Neuhauser, 2007; Persson et al., 2021; Staines et al., 2015). In international comparison, Sweden has deficiencies in the person‐centeredness of care but shows good results with few persons who forego care for financial reasons and a high degree of digitalization (Vard‐ och omsorgsanalys, 2021). Based on a previous study on contact patterns and costs (Lind et al., 2022), the aim of this study is to explore lived experiences in a population of persons living with MS in a Swedish context, representing different contact patterns with healthcare.

## 2 MATERIALS AND METHODS

This qualitative interview study draws on 10 semi‐structured interviews with persons living with MS underpinned by a pragmatic worldview (Johnson et al., 2007). The interviews took place in the first quarter of 2022.

### 2.1 Recruitment and data collection

**FIGURE 1:**
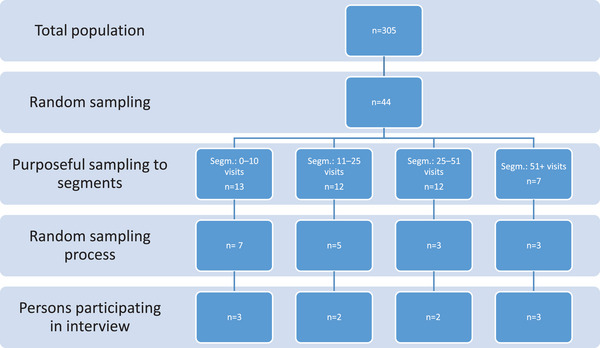
Schematic of the different phases of the sampling process for the interview study, based on a former quantitative study that identified four segments of utilization of healthcare services (Lind et al., 2022).

Participants were recruited based on a quantitative study involving all persons who received the diagnosis of MS (ICD‐10 code G35) in a population‐based healthcare system in southeast Sweden between January 1, 2018 and September 30, 2019 (Lind et al., 2022). Four different patterns of healthcare utilization (segments) were identified based on the number of visits patients had in the healthcare system (Figure 1). The sampling process in this study included both purposeful (Emmel, 2013) and random sampling (Robinson, 2014) from the total population (*n* = 305) (Figure 1). A random sample in the first step was used as a tool to narrow the total population from the total *n* = 305. The criterion for the purposeful sampling was having all four segments represented in the study, while the second random sampling process was a tool to select participants within the segments. The first random sample from the total population resulted in 44 persons, which were purposefully divided into groups based on the four segments of healthcare utilization, so as to have all segments represented in the study. Within the segments, a random number sample was made where every third person received a letter with written information about the interview study along with an informed consent form. If several persons within one segment declined participation, the number sampling process was repeated within that segment until all segments were represented with at least two persons. After the written informed consent form was signed, the persons were matched to the relevant segment in the database. Ten persons chose to participate. Eight persons declined or did not answer the phone call or letter.

A semi‐structured interview guide was developed based on earlier work within an international improvement collaboration (Oliver et al., 2019) and earlier work exploring lived experiences of persons in professional and leadership programs (Johnson et al., 2021; Persson et al., 2021). One structured question on health assessment of the state of health the last year was included, measured on a five‐point scale from very good to very bad.

The interviews were conducted by two members of the clinical team, with methodological support in conducting research interviews from the research group. Two pilot interviews were conducted to develop skills and understanding of interactions, and some questions were rephrased in coproduction between the persons with patient experience, professionals, and researchers.

Seven persons chose to be interviewed through a digital video tool, recorded as video files, and three chose face‐to‐face interviews at the hospital, which were audio‐recorded. The interviews lasted on average 47 min and in total 390 min (with a variation from 22 to 55 min) were recorded and transcribed verbatim.

### 2.2 Data analysis

Data were analyzed using inductive thematic content analysis to identify patterns and meanings across the data (Braun & Clarke, 2006). The reflexive analysis followed the six phases proposed by Braun and Clarke (2006, 2019). All transcripts were read through to get a sense of the whole and were checked against the audio files. The analyses were conducted manually, highlighting data extracts and creating initial codes. The initial coding scheme was documented in a Word file with tables and text. Codes were collated into initial themes. The themes represented patterns within the data at a semantic level. A reflexive process of going back and forth between data extracts, codes, subthemes, themes, and transcripts was ongoing during the analyses. To understand patterns and variation among the data sets, themes and related codes were connected to data extracts from the different interviews. Two researchers (Sofia Persson, Jonas Lind) read all the interviews, and one researcher (Sofia Persson) performed initial analyses and created coding schemes. Initial coding schemes, with themes, codes, and data extracts, were analyzed together with one experienced qualitative researcher without relation to the clinic (Ann‐Christine Andersson) in a reflective process (Braun & Clarke, 2006). The analysis was presented and discussed in the research group and the clinical team with the intent to refine each theme as well as the overall narrative in the analysis.

## 3 RESULTS

### 3.1 Interviewee demographics

Ten individuals participated, five men aged 43−67 and five women aged 30−71. Nine were married or described having a partner. They represented different livelihoods, being employed full time (*n* = 5), on parental leave (*n* = 1), on part‐time sick leave (*n* = 1), in early retirement due to illness (*n* = 1), or in old‐age retirement (*n* = 2). The health assessment rating resulted in two very good, five pretty good, two reasonable, and one rather bad.

### 3.2 Analysis

The analysis generated 4 overarching themes with 12 subthemes (see Table 1). In what follows, each theme will be presented with its associated subthemes, illuminated with quotes (Table 2).

**TABLE 1:** Overview of the results’ themes and subthemes.

Themes	Subthemes
A. Perspectives on life and health	Relations with others Personal attitudes Future perspectives
B. Influence on everyday life	Interests and activities Symptoms and consequences Aspects of medication
C. Relations with healthcare	Diagnosis confirmation One's individual requirements Building knowledge
D. Shared healthcare processes	Access to care Planning and coordination General perceptions

**TABLE 2:** Participant quotes.

A1. Relations with others
“I have been pretty open about this MS thing towards those who have been interested and want to know.” (1) “I didn't want to tell my grandmother and so on that I was sick. They always become so upset.” (4) “It's not a secret anywhere to anyone. If someone asks, I'll tell them, and if there's someone who I think should know they'll get to know it, but the way I'm feeling there isn't anyone who needs to know.” (6) “The family knows about it, my family, my children know about it, obviously. Then my boss knows about it at work because I do need to take time off sometimes, but otherwise, I haven't talked about it at work with anyone besides my boss. I think I'll keep quiet as long as it doesn't show. One day, if I was to become worse, if I get worse before I retire, then I may as well say something so that people won't wonder what's wrong with me.” (7) “Surely I felt the MS at times then, and sometimes I dragged my leg at work and felt that it was hard, having a relapse or something, but you blamed it on something else … so nobody knew that I had MS.” (10)
A2. Future perspectives
“I see a bright future ahead of me. You have to. Most things can be solved in some way. I can put on a diaper under my pants in case I wet myself, and I can, like, whatever it is, avoid a lot of things that I know I cannot manage [to do]. But precisely the freedom and movability and such means a lot to me.” (1) “You think about the future. You don't know how it's going to unfold or what is going to happen, so that's been a little tough. I didn't envision those who only have a slight limp. But I met them at the MS class. So, I let go of that worry. In the beginning it was really hard because then it was like, shit, now I'm going to end up in a wheelchair. I'm going to become paralyzed and I will become, but that was like, I only envisioned the worst MS patients. I didn't see those who may have had, who haven't had any treatment their whole lives, but still walk well and only have a slight limp. I met those at this MS class and then, but shit he's doing, like, really well.” (2) “I'm not worried about anything. A doctor told me when I was there at some point that the way things are looking, with a little bit of bad luck, you'll have a cane when you're 75.” (6) “If it stays like this I'll have a good life, of course … but if it doesn't, then you don't know what is going to happen … you don't know, because I know some people who have MS and they found out about it when they were 30 like me, and, for them it's just gone downhill and become worse and worse, so you're a little scared that it'll be the same, but not everyone is the same after all.” (9)
B1. Symptoms and consequences
“That type of activities I have to opt out of, of course. I stand on the side at ice hockey, it's so cold, so there are my wife and kids skating, and I get to stand there and watch …” “Then, as always, of course you're tired at times and such, and then I don't know, so to speak, if it has to do with my medication or with the disease, or if it's just a general thing, you can be tired in the spring and you can be tired in the fall, yeah, a little like that. But not that I've had to be away from work, it hasn't been like that.” (1) “I have a good everyday life, except when I become very tired. I have to adapt activities to how tired I am, and when I clean, for example, I need to rest for two days.” (2) “I feel healthy—I even forget when you ask about symptoms.” (4) “There at home, everything is affected, truly everything. Sex life, socializing and all the other friends and everything like that you had. Now I have a few left but not that many. So everything. There's probably nothing that hasn't been affected … It has cost me a completely normal life, simply put … I've done all those things, and nothing like that is left.” (5) “I slip away at times when there are physical challenges in team‐building and stuff like that at work.” (7) “When you're standing up and cooking, you have to sit down often. When you're walking you have to lean a lot against the walls and tables and such, you support yourself to keep your balance and …” “It's that you can't, can't walk that far, I can't ride a bike of course or meet friends in the same way because you can't manage to move in the same way, so … // … Of course my pay and everything has become lower … you've become stranded a lot. There's a difference, of course, between a sickness pension and a regular salary.” (10)
B2. Aspects of medication
“I've understood that some experience these flushes as being so discomforting that they choose to discontinue their treatment and take a different medication instead, and others, like me, out here if you say so, hope that it gets better. They don't think that it's that bad so that it, like, warrants any major action, but then again. What, is this dangerous, or does it matter, so to speak, that I turn bright red and it stings and itches and stuff like that? Is this dangerous for me? Of course I have, and there hasn't been a proper answer …” (1) “I am medicated once a month and it has evolved a lot. Before I had to be there an hour in advance, an hour afterwards and then the treatment took an hour, so that's how it is. Now I get there and come and go and it's like, there's never anything odd, so, yeah. No, it works really well.” “… // … like some time I was there and got Tysabris and I thought like this, yeah well then it was 3 hours in the beginning. Well, then I thought, like, I get 3 hours to myself, just like that. Just being, and, like, all that stuff, and then this other girl came in next to me and she had bought this really big pastry because it was such a pity for her that she had to be there 3 hours. Well, a little like that, and I look at it this way, it's an opportunity for me to just kind of unwind right then. No, then I thought like this, I don't see it as a problem, so I think I'm probably positive in that way.” (3) “Well, my illness and medication, there have been some question marks. What it does and what the difference is. But precisely how it affects me, because it does affect me a little.” “I had to stay there for my first pill and they checked my pulse and it went rather well. I'm alive, anyway … // … So, actually I don't really know what this medication actually does to me as long as it, well, I have no need to know what it actually does to me as long as I know that it is a good medication.” (6) “[It] was more in the beginning when they were to find the right medication, and some were about to commit suicide in one treatment. It wasn't good for me at all, but luckily I made it. I've had four different ones, it's the fourth type I have now. The other three were completely worthless. You have to accept this journey. They start with the simplest and cheapest one, of course. It's about taxpayers’ money and I get that.” (7) “Well, it's medication you have to pick up every year, quite a large sum to get your medication. So, every year you have to pay 2000 kronor to be able to get your medication, so that's it. It's not cheap.” (9)
C1. Diagnosis confirmation
“I logged onto my journal on 1177 [the Swedish healthcare system's dedicated website] to check another test, then I saw the MR result … had I not seen it perhaps it would have gone much better. I saw suspected MS … I panicked … // … I barely remember anything … I was completely messed up in my head.” (2) “They started talking about that it could be MS and I was pregnant and didn't know what was going to happen with my body and all that, incredibly frustrating and mentally stressful in every way … // … I'm sure I would have been able to get support from someone to talk to within the healthcare system then, but I chose to go through another [provider] at the time. I guess you just need someone to talk to, maybe not just your family, but somebody else beyond that … // … So, for me that was very, very valuable. I missed anybody even addressing the question of counselling …” (3) “I didn't know whether I would go home and die or what it was … // … I got the healthcare center physician's phone number who said that either you have blood cancer or you have MS, and it can't be blood cancer because then you wouldn't be sitting here.” (4) “In the beginning I didn't get any support from healthcare. They probably need to get their act together, if they haven't done so already … // … I got the verdict [thrown] right in the face and then nothing more happened. I sat down in my car and it all went black. I might as well have killed myself in a car accident on my way home because then I was close to trying, thought about whether I should aim at a truck or not. It's horrible information [to receive] so you shouldn't just let a person go like that. You should have been given some information and stayed there for an hour or so. What comes up in your mind are people in wheelchairs and the final stage … // … It's a crisis, a life crisis.” (7) “So, when I got my diagnosis, it was really, my life was a little, well, scattered in a way. I got the diagnosis at an appointment alone with the doctor. I don't know how I made it home to my wife with the car after that, because it all came crashing down … It wasn't really good.” (8)
C2. Building knowledge
“There's a lot you can read and hear about when it comes to others’ fates and such, but I've tried to avoid this but yeah, everyone's different … // … I want to avoid meeting others with MS … but of course, I mentioned this before, that I probably wouldn't feel great meeting a group of people who walked with crutches or sat in wheelchairs or could in other ways be in very bad shape because of this, because then I'm reminded of something that I don't really want to be reminded of. Then again, with those who are in a situation similar to mine, those I could have an exchange with. What do you do in everyday life to alleviate these side effects or whatever it may be? Now during the pandemic situation it hasn't been the time either for this type of group exchange and maybe I don't really think that it would suit me to have to go to some kind of place and meet other people in that way. I wouldn't really be comfortable in that situation.” (1) “In the beginning there was this group, well, it was about brain fatigue, how to think and stuff like that. For me it was really hard then because I didn't want MS to, well that my illness would take over my life. It was like, should I have to change my whole life because I've gotten …? You don't really have quality of life if you're to live according to these conditions.” (2) “In the beginning maybe you read online. Now today I don't read that much. I wrote for XX XX on their blogg for a while, about what it is like to have MS and things like that … but in the end I didn't have anything to write about any more … // … I never felt that I had the need to talk in a group with only people who have MS. I think that many can experience that they have a form of support there. I felt that I had many others to talk to. And then illnesses are different, too.” (3) “I don't really have any contact with others and I don't want that, either. No, then I would feel ill. No but, it's just that then you see that many others are worse off than you. I have, there aren't that many who are doing so well, and then I almost feel guilty because, wow I'm doing really well. Those poor others, a little negative instead, I think. That's me.” (4) “I could have had contact with others with MS if I had wanted to. We've had associations, local associations. They have been shut down now because there were no more members. It was rewarding to meet others, it was pretty good, you could meet others and see how they functioned and so on.” (8)
D1. Planning and coordination
“I would have liked to have more clarity … that now you go every third month and take tests for the time being, or for one year we'll book this … Now I can go and wonder, is it time soon? A detailed, concrete plan isn't really anything I would have expected, but it would have made things easier … // … This plan then, in general, both in working life and in school, there are all these individual development plans. That would have made things easier for me.” (1) “I'm the type of person who wants to know things. Okay, this is about my illness, so it's about my body. I would really like to know what is going to happen. What's the plan? What will it look like in the future? What is my treatment going to be? How often do you need to see a doctor, really? Is it once a year, every other year?” (2) “There's a care plan for me, I know that. I don't know what it looks like, but on the other hand it will be a fun lesson to see what it will look like, given that I am the way I am.” (5) “It would be nice to have more information … now we'll do this or that. I don't get any of that here at the hospital, something like, now we will, now we will do this. … Now you'll get this or something like that.” (8) “And now I don't really know which doctor I'm going to get. I find that a little uncertain for the future. I would kind of like to have clarity on who I am going to have … that I can contact this doctor if there is anything. And that's like, I don't know anything, what's happening. I used to have an annual visit but that was through some sort of phone contact, because I don't want to go just like that, either, but you want to know more about what is happening there, you know … so that you don't stand there without …” (10)
D2. General perceptions
“By and large I have positive experiences of healthcare. Then I don't have … it's a personal responsibility for the individual, too.” (1) “I'm very content. Healthcare is already good, first of all. Secondly, it can always be better. Simply be sensitive. Listen to the patients, take it in [and] weave it together in one way or another …” (5) “I think that healthcare in general is good. The thing that could work better is this connection between the primary care center and the hospital.” (7) “In general I'm content, very content. It's just small details you get annoyed with. I have been treated well, that I have to say. The care I have sought, or help, has been really good. You just need to get past the primary care …” (8) “Well, I'm on it myself, so that … yeah … yeah … I think I get what I want, anyway … it depends, but you have to be able to speak up yourself, too.” (10)

### 3.3 Perspectives on life and health

In relations with others, family was most important. Support from the partner/husband/wife was emphasized and included practical, emotional, and disease‐related aspects. Practical support could include carrying bags, cleaning, and driving one to appointments. Emotional support was sharing feelings, worries, and struggles in everyday life. Other areas of support were helping in observing the progression of symptoms and assisting in medication procedures such as injections. The possible burden for partners/family was sometimes acknowledged. Most of the interviewees chose to be open about having MS in their networks and workplaces. Motives for telling were practical reasons or explaining visible symptoms. Some were more restrictive about whom they told. Motives for not telling could be that you just did not need to tell when you did not appear or feel sick, wanting to protect relatives from sadness, or not wanting colleagues to know (Table 2, A1). Reactions from others ranged from being met with empathy and understanding to experiencing a lack of understanding, especially when no symptoms were visible.

In personal attitudes, being a positive person was expressed by a majority of the individuals as an important approach to handle living with MS. Even though living with MS played different roles for different individuals, they described using their own resources to cope with limitations and symptoms, as well as appreciating their current abilities. Feelings of gratitude and being fortunate were sometimes linked to the awareness that things could be worse. This entailed realizing that you could have had much worse symptoms or that you could have gotten another, even worse disease.

Hopes and fears were described concerning the future. Hopes regarded the disease not getting worse, and their own capacity to overcome difficulties that might arise. Fears were mostly concerned with the loss of movement, becoming paralyzed, and becoming dependent on a wheelchair. Clear information from the physician on future abilities reduced uncertainty and worries. And having others with MS around could increase both hope and worries (Table 2, A2).

### 3.4 Influence on everyday life

All interviewees described engaging in some form of physical activity to promote their own health and well‐being as an important aspect of everyday life. A majority handled most of their exercise on their own. Exercise intensity was handled differently, with some limiting intensity as they worried about how intense training could affect the disease, while others described very intense training sessions. Some described careful diets and balancing activities and rest as important parts of everyday life.

Symptoms described were sensory impairments, motor impact with the loss of strength and balance impairments, urine leakage, and fatigue. Consequences varied from no negative consequences at all to challenging consequences in all areas of life (Table 2, B1). Life areas affected by the symptoms were leisure activities, family life, work, and relationships. The MS diagnosis had influenced career choices for some individuals, such as leaving a manager position for a less stressful work environment and deciding not to pursue a career. Some described their work capacity as limited or non‐existent due to MS, while others described no limitations at all.

The medication's role in everyday life depended on whether the person had ongoing medication, the type of medication they had, and the side effects experienced (Table 2, B2). For some, medication and thoughts about it played a big part in everyday life, while for others it did not. Several described experiences of the processes with medication changes and adjustments to find the right medication as difficult, with a negative impact on well‐being and function.

Financial perspectives regarding medication included that they were a cost for society and healthcare, and had effects on one's own private finances. Thoughts that economic interests in healthcare affected the process of trying out medicine were expressed. Living with concerns, worries, and questions regarding how long‐term medication and side effects affected the body and health over time were areas that were described and experienced as not satisfactorily answered by the healthcare system.

### 3.5 Relations with healthcare

Most persons received the diagnosis confirmation during a visit to the physician at the neurology department and experienced a lack of support from healthcare at the visit. The visits in general were experienced as too short, with no support of one's own (family member/partner) present, and left the individual having to handle the shock alone afterward. The feelings described by most were as if their life collapsed temporarily and they visualized life in a wheelchair. One person described suicidal plans on the way home after the confirmation (Table 2, C1).

Resources and needs in the relations with healthcare were central in the subtheme of one's individual requirements. The visits to physicians were the most important part of the relationship. Some described coming prepared with bullet points and questions, determined to carry through their own agenda at the visit, while others perceived that the visits were predetermined from the healthcare perspective without any time to address what was important to them. How often and in what form the individuals wanted to have contact with healthcare varied. Both digital and physical visits could be suitable depending on the situation and individual needs. Digital consultations were experienced as positive if the individuals did not have significant changes in physical symptoms, if the effort of the visit otherwise would increase the burden of illness/tiredness for them, or if they wanted to keep away from others due to the risk of infections. These contacts could bring a sense of relief that everything was going well, but also leave the individual with unanswered questions or feelings of not being listened to.

Aspects of knowledge building on MS ranged from not wanting any information to wanting to have the same level of knowledge as the professionals in the clinic. Sources of information used were networks (online or face‐to‐face), contacts with healthcare, and internet sites. Searching for information on the internet was described by a majority. Some described that internet forums were mostly populated by persons who were very ill, which could generate a negative view of living with MS. Others could find a sense of coherence, feeling that they were not alone in handling struggles. Sometimes the physician was an important partner in reasoning about information and knowledge development, but it could also be confusing to get different information from different healthcare experts. A majority did not want to meet others with MS face‐to‐face for knowledge development. Reasons were not being comfortable in a group situation, feelings of guilt if you were not as affected as others, not wanting to be associated with or identified as a person with MS, or wanting to avoid being reminded of things about the disease that you did not want to think about (Table 2, C2).

### 3.6 Shared healthcare processes

Access to care regarded how the persons experienced getting in contact with healthcare. Regarding their MS symptoms, telephone contact with the clinic was the most common way. The relational aspect of talking to someone was highlighted. Getting to the right competencies was sometimes delayed, such as getting to see the urologist for urinary incontinence, or still having to go through primary care even though the person self‐assessed that their condition needed competencies at the hospital.

Planning and coordination regarded the design of healthcare services and treatments. Coordination between caregivers varied; some experienced well‐functioning coordination when having contact with several clinics and/or primary care, and some experienced no coordination at all. A majority of the persons wanted clearer information and a plan regarding their annual visits and examinations. Not knowing generated insecurity and worries for some, that they were forgotten by healthcare, and reflections on the right to have access to information, planning, and processes regarding their own lives were described. Comparisons were made to development plans at work or school and similar plans were suggested to be useful for healthcare contacts (Table 2, D1).

General perceptions regarded the individuals’ views on healthcare as one system. A majority expressed a positive overall view. Positive experiences highlighted were concerned with access, help and support, and being treated well. Even individuals describing isolated disappointments or errors from healthcare were positive in their overall experience. The shared responsibility in the relation between the persons and healthcare professionals for value and outcomes was highlighted. Improvement areas for healthcare in general were that they should be more responsive, listen to the persons with patient experience, and improve cooperation and coordination between primary care and hospital care (Table 2, D2).

## 4 DISCUSSION

Lived experiences of persons living with MS have been explored in different populations and contexts (Borreani et al., 2014; Galushko et al., 2014; Hunter et al., 2021; Methley et al., 2017). This study in a Swedish context identified four themes that regarded the patients’ own perspectives and context, as well as medical and healthcare‐related aspects. Patterns of more shared experiences were found, for example, in the diagnosis confirmation, future perspectives, and planning and coordination. More individual experiences appeared concerning, for example, relations with others, one's individual requirements, symptoms and consequences, and knowledge building. The complexity of the MS disease has often been highlighted as the reason for the complex and diverse needs of the population (Borreani et al., 2014; Galushko et al., 2014; Morley et al., 2013). This diversity and complexity was also found in our study, as MS played different roles for different individuals. In addition, other factors such as close relations, aspects of medication, personal attitudes, and relations with the healthcare system were examples of other important aspects of the person's lived experience. These findings can relate to studies describing medical, social, and personal factors as mechanisms of the patient's experience, influencing the balance between the patient's workload and capacity to handle life with the disease (Boehmer et al., 2016a, 2016b). All such factors are dynamic and change over time with one's disease and life journey. This highlights the need for the adjustment of healthcare services, coordination, and planning not only concerning MS but also the whole life situation of the person. One example were contacts with healthcare, where MS, the healthcare system, and other aspects of lived realities outside healthcare influenced how, when, and where (digital or physical) consultations would be most valuable for the different individuals. This suggests a need for more coproduced planning and design with more diverse service options and content to facilitate person‐centered care. Persons living with MS can also have other temporary or chronic conditions with treatments over a shorter or longer period in different parts of the healthcare system (Lind et al., 2022) that need coordination. These findings in the MS population is similar to the Swedish healthcare system as a whole where challenges to anticipate and respond to patients as individuals with particular needs and preferences together with inadequate coordination of care between different healthcare providers have been described (Docteur & Coulter, 2012).

The visits with the diagnosis confirmation were a very difficult time for most persons, with experiences of lack of support from healthcare, similar to what was found in previous studies (Hunter et al., 2021). Involving the person's close relations such as partner/husband/wife in the visits, offering support, and showing a deeper understanding of how the confirmation affected the person's views on life and the future were suggested as improvements for healthcare to meet the needs of the population.

We found that websites often were used if the individuals wanted to connect with other people's experiences, gain knowledge, or find other information on MS and medications. Similar patterns regarding knowledge generation have been described for Parkinson's disease where the main source of disease‐specific knowledge was found by the persons themselves online (Riggare et al., 2019). A minority wanted to engage in face‐to‐face groups with other persons with MS, which is in contrast to another study describing MS support groups as central for assistance and friendship (Haubrick et al., 2021). Instead, the most important support in our study came from close relations. The importance of personal integrity regarding what to share with whom, and in what way, was highlighted. However, an effect of the COVID‐19 pandemic during the study on the patient's willingness to take part in in‐person meetings cannot be ruled out.

In our sampling process, a larger proportion of persons declined participation in the segments with few healthcare visits. Persons in this segment had little contact with healthcare in general and could perhaps be less likely to engage in requests from healthcare. Aspects of who says what, when, with what gender, age, or background were not the focus in this study, but rather the exploration of patterns in the total population.

Having persons from the clinical team conduct the interviews was a strength when it comes to the pre‐understanding of the population and setting, but a limitation due to the risk of bias, power, and the interviewees feeling dependent and therefore reluctant to be open about difficulties in contact with healthcare. These limitations were reflected on beforehand, during the analysis, and in relation to the results. In the interviews, patterns of both similarities and differences in the population as well as positive and negative aspects of healthcare were found. Therefore, the team chose not to add additional interviews or change interviewers. The qualitative research interview is never neutral, and issues of power and other factors that both enable and complicate matters are always present (Kvale et al., 2014). The point is not always to eliminate issues of power but rather to reflect on the role power relations have in the knowledge gained (Kvale et al., 2014), and our findings must be viewed in light of this. The knowledge claim of this study is not to give a complete picture but rather explore examples of patterns within a population representing different contact patterns with healthcare. Dialogues on the analysis and results within the clinical and the research team strengthen the reflexivity in the study, giving both inside and outside perspectives with different competencies and prior understandings (Lincoln & Guba, 1985). The video or face‐to‐face setting for the interviews was chosen by the participants and we observed no differences in the interviews or length between the different settings.

## 5 CONCLUSION

This study provides information about both shared and individual patterns of lived experiences in a population living with MS in a Swedish context. The findings suggest a need for a more diverse and coproduced development of healthcare services to meet diverse needs in the population with greater acknowledgement of the person's lived experience, including consideration of the complexity of the disease, personal integrity, and different ways of knowing. The need for healthcare in general to be more responsive and listening in to the patients was emphasized and underlines the importance of coproduction.

Findings from this study will be further explored together with other quantitative and qualitative data regarding, for example, contact patterns, MS type, medication, and quality of life assessments to develop a deeper understanding of associated factors and facilitate service development. Coproduction in the context of service development in MS care also needs to be further explored in improvement initiatives and research.

## AUTHOR CONTRIBUTIONS

All authors were involved in the design and the discussion of analyses and results and contributed to the writing process of the article. All authors read and approved the final manuscript. In the thematic analyses, Sofia Persson and Jonas Lind read all the interviews, and Sofia Persson performed initial analyses and created coding schemes. The initial coding schemes were analyzed by Sofia Persson and Ann‐Christine Andersson together. Bertil Lindenfalk supported the persons conducting the interviews. Boel Andersson Gäre was involved in design, analyses and result discussions as well as the writing process.

## CONFLICT OF INTEREST STATEMENT

The authors declare no conflict of interest.

### PEER REVIEW

The peer review history for this article is available at https://publons.com/publon/10.1002/brb3.3104

## Data Availability

The datasets used and analyzed during the current study are available in anonymized form from the corresponding author upon reasonable request.
